# Intraocular Pressure Changes during Femtosecond Laser-Assisted Cataract Surgery: A Comparison between Two Different Patient Interfaces

**DOI:** 10.1155/2019/5986895

**Published:** 2019-09-25

**Authors:** Chiara De Giacinto, Rossella D'Aloisio, Alessandro Bova, Tommaso Candian, Alberto Armando Perrotta, Daniele Tognetto

**Affiliations:** Eye Clinic, Department of Medicine, Surgery and Health Sciences, University of Trieste, Trieste 34129, Italy

## Abstract

**Purpose:**

The aim of this retrospective cohort study was to evaluate intraocular pressure (IOP) changes during femtosecond laser-assisted cataract surgery (FLACS) using two different patient interface systems.

**Methods:**

116 eyes of 116 patients scheduled for cataract surgery were divided into 2 groups: group 1 (61 eyes) and group 2 (55 eyes) underwent FLACS using Catalys Laser with fluid interface (liquid optics interface, LOI) and LenSx Laser with curved interface and soft contact lens (SoftFit), respectively. IOP was assessed using a portable rebound tonometer (Icare®) preoperatively, after docking, immediately after surgery, at one and seven days postoperatively.

**Results:**

In group 1, the mean IOP (±SD) was 14.1 ± 0.4 mmHg before surgery, 33.2 ± 1.1 mmHg after docking, and 21.4 ± 0.9 mmHg immediately after surgery. In group 2, the mean IOP was 13.8 ± 0.4 mmHg before surgery, 24.2 ± 1.4 mmHg after docking, and 20.2 ± 1.2 mmHg immediately after surgery. After the docking procedure, a statistically significant increase in IOP from the baseline was found in both groups (*p* < 0.001). Moreover, no statistically significant difference in IOP measured at 1 and 7 days postoperatively was observed compared with the preoperative values (*p* > 0.05) using both laser platforms. No intraoperative and postoperative complications were observed.

**Conclusions:**

FLACS suction phase resulted in a transient increase of IOP in both groups, especially with the LOI system, and it is probably related to the greater pressure of a suction ring and suction generated through the vacuum, independently from the effect of femtosecond laser itself.

## 1. Introduction

Femtosecond laser technology, firstly introduced as a new technique for creating lamellar flaps in laser-assisted in situ keratomileusis (LASIK) in 2001, has been rapidly developed for cataract surgery, showing several advantages in different surgical steps, such as corneal incisions, anterior capsulotomy, and lens fragmentation [1, 2].

All FLACS systems rely on interfaces for the docking phase, which is one of the most delicate steps, allowing good suction and the final success of laser treatment [1]. Docking has three functions: the optical coupling allowing efficient delivery of the laser beam into the ocular tissues, the ocular mechanical stabilization during laser application, and the accurate acquisition of bi- and tri-dimensional images ensuring the correct position of the corneal incisions [3, 4].

However, an increase in IOP during the docking step has been previously reported because of corneal compression during the applanation process [5–7]. The first published studies that compared two different femtosecond lasers have evaluated IOP changes in vivo and ex vivo animal models, describing a higher increase in IOP using the flat applanation interface in comparison with the curved one [5–7].

Fluid-filled interfaces have been developed to cope with applanation interface-related rise in IOP and have shown to cause less corneal folds and a lower IOP increase than curved contact lens interface [8].

The liquid optics interface (LOI) of Catalys® Laser, similarly to ultrasonic examination devices, uses water immersion to minimize the impedance mismatch between the transducer and the eye [9]. LOI permits less pressure on the cornea as there is no direct force or deformation of the corneal structure [8].

On the other hand, LenSx® Laser has a one-piece patient interface connected to the counter-balanced laser objective head and is characterized by a curved applanation interface combined with the most recent upgrade of soft lens-assisted interface (SoftFit™) [10].

The aim of this study was to investigate IOP changes during and after FLACS in human eyes which underwent surgery performed by the same surgeon, comparing two completely different patient interfaces: Catalys® Liquid Optics Interface and LenSx® SoftFit™ interface.

## 2. Materials and Methods

This retrospective cohort study included 116 eyes of 116 patients who underwent FLACS and intraocular lens (IOL) implantation performed by the same experienced surgeon (D. T.) at the Department of Medicine, Surgery and Health Sciences, University of Trieste, Italy, between December 2015 and October 2017. This retrospective observational study adhered to the tenets of the Declaration of Helsinki, and our Institutional Review Board approved the retrospective consecutive chart review.

The inclusion criteria were the following: medium lens opacities according to the Lens Opacities Classification System III (LOCS III) [11], good pupil dilation (≥5.0 mm), and age above 18 years.

The exclusion criteria were the following: glaucoma, corneal opacities, previous surgical treatment within 6 months, and ocular inflammatory conditions.

Informed consent was obtained from all eligible subjects for the use of their data.

A total of 61 eyes were treated with Catalys® Precision Laser System (Johnson & Johnson, Santa Ana, CA, USA) with LOI (group 1) and 55 eyes with LenSx® Laser System (Alcon, Fort Worth, TX, USA) with curved contact lens SoftFit™ interface (group 2).

### 2.1. Patient Interface Docking Procedure

In group 1, the Catalys® Precision Laser system with the LOI module used a two-piece process: a suction ring that has contact only with the sclera (patient interface dimensions: an outer diameter of 22.3 mm and an inner diameter of 13.5 mm) and a disposable lens linking the suction ring to the surgical system. Firstly, the suction ring was located perfectly central on the sclera of the eye. After some minimal adjustments, the suction ring vacuum was created leading to the connection between the suction ring and patient eye. Secondly, a balanced salt solution (BSS) was used to fill the suction ring volume. Finally, the suction ring was adjusted and connected to the laser head docking. Regarding group 2, the LenSx® system used a single-piece patient interface (patient interface dimensions: an outer diameter of 19.8 mm and an inner diameter of 12.5 mm) that was attached to the machine and then docked to the eye using the joystick and the video image on the graphic user interface. After patient docking, both LenSx® and Catalys® platforms performed OCT imaging of the eye which is 2D and 3D, respectively. The images were analysed for treatment planning and for the final steps of the femtosecond laser treatment, such as anterior capsulorhexis, nuclear fragmentation, and corneal incisions.

### 2.2. Intraocular Pressure Reading Protocol

The day before surgery, all patients had a complete ophthalmic evaluation including best corrected visual acuity examination, slit lamp biomicroscopy, IOP measurements assessed with a portable rebound tonometer (Icare®, TA01I, Icare Finland Oy), biometry, central corneal thickness, and anterior chamber evaluation.

IOP was measured with Icare® just before the docking phase (5 minutes before), after the docking procedure (just after that the eye was undocked, about 1 minute later), and just after the end of surgery, at 1 day and 7 days postoperatively, during follow-up visits. Each time, the average of 5 IOP measurements was considered and registered on our electronic medical records. All patients were placed in the sitting position in the operating bed for the pressure reading procedures. Before surgery, all eyes were anesthetized with oxybuprocaine hydrochloride 0.4%. In addition, phenylephrine and tropicamide 10% + 0.5%, tropicamide 1%, and anti-inflammatory eye drops of diclofenac sodium 0.1% were used before surgery as well. After the patient was docked to the system, the OCT imaged the anterior chamber, and the system created a 3D treatment plan with the Catalys® system and 2D with the LenSx® system. After laser application and removal of the suction, manual surgical cataract procedure and IOL implantation in the capsular bag were performed in the same operating room.

### 2.3. Statistical Analysis

Continuous data were expressed as the mean ± standard deviation (SD). Statistical analysis was performed using IBM® SPSS Statistics v 20.0 software (SPSS Inc., Chicago, Illinois, USA). Repeated measures ANOVA with linear trend analysis were performed to evaluate the effect of surgical phases on IOP values. Contrast analysis was performed to evaluate differences of each parameter from the previous measurement. A *p* value less than 0.05 was considered statistically significant.

## 3. Results and Discussion

### 3.1. Results

A total of 116 eyes of 116 patients were considered.

The demographic and clinical characteristics at the baseline are reported in Table 1.

No statistically significant difference was found between the two groups in terms of clinical cataract grading, preoperative IOP levels, anterior chamber depth, central corneal thickness, and axial length (Table 1).

**Figure 1:**
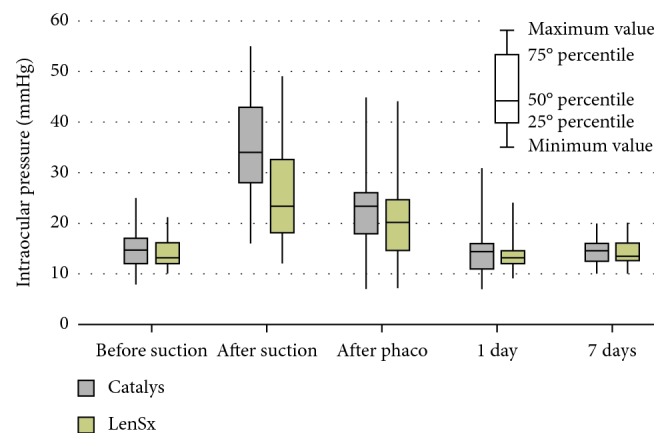
Intraocular pressure values measured at different time points during FLACS performed with two different devices: Catalys and LenSx. The box is determined by the central mean as well as the 25^th^ and 75^th^ percentiles. The whiskers represent the minimum value (0^th^ percentile) and the maximum value (100^th^ percentile).

Group 1 was composed of 61 eyes with a mean age of 72.4 ± 7.6 years (ranged between 47 and 89), and in group 2, 55 patients with a mean age of 71.5 ± 13.9 years were evaluated (ranged between 23 and 89). In group 1, the mean IOP (±SD) was 14.1 ± 0.4 mmHg before surgery, 33.2 ± 1.1 mmHg after docking, and 21.4 ± 0.9 mmHg just after surgery. In group 2, the mean IOP was 13.8 ± 0.4 mmHg before surgery, 24.2 ± 1.4 mmHg after docking, and 20.2 ± 1.2 mmHg after surgery (Table 2, Figure 1).

In both groups, a statistically significant increase in IOP after the suction procedure was found from the baseline (*p* < 0.001; Table 2, Figure 1). Moreover, in both groups, the IOP measured at 1 and 7 days postoperatively did not show any statistically significant difference with the preoperative values (*p* > 0.05; Table 2).

A statistically significant difference between the two groups in terms of rise in IOP was found only after the docking phase with a higher value in group 1 (Table 2).

In group 1, the mean total suction time was 2 minutes and 50 seconds ± 54 seconds; the laser capsulotomy was completed in 1.5 seconds using the following treatment parameters: pulse energy range 4-5 *μ*J, 4.9 mm diameter capsulotomy, 5 *μ*m horizontal spot spacing, and 10 *μ*m vertical spot spacing; the capsulotomy needed little energy, only 0.7 J in each eye. The mean lens laser fragmentation time was 48 seconds ± 27 seconds. The lens was segmented into quadrants or quadrants softened patterns with the following treatment parameters: grid spacing range 350 *μ*m–800 *μ*m, segmentation repetition range 5–10, 10 *μ*m horizontal spot spacing and 40 *μ*m vertical spot spacing, anterior pulse energy range 8–10 *μ*J, and 10 *μ*J posterior pulse energy. The mean total energy lens fragmentation was 12.8 ± 4.9 J.

In group 2, the mean total suction time was 2 minutes and 45 seconds ± 34 seconds; the laser capsulotomy was completed in 2.5 seconds using the following treatment parameters: pulse energy range 7-8 *μ*J, 4.9 mm diameter capsulotomy, and 5 *μ*m tangential and layer spot separation; the capsulotomy needed only 0.6 J of energy in each eye. The mean lens laser fragmentation time was 46 seconds ± 20 seconds without any statistically significant difference if compared to group 1 (*p*=0.67). The lens was segmented into cylinders or matrix grid patterns with 12 to 13 *μ*J posterior/anterior pulse energy.

Laser-assisted cataract treatments were successfully performed in all cases. No intraoperative and postoperative complications were observed, including suction loss during the laser treatment or posterior capsule rupture, and no adverse events occurred. Furthermore, no patient reported amaurosis.

IOL implantation in the capsular bag was performed in all cases.

### 3.2. Discussion

As previously described, a rise in IOP during the docking phase in FLACS was related to the use of a suction ring and to the corneal applanation during cone device coupling. Indeed, advances in interface technology have been developed, and new patient interfaces with and without the corneal applanation procedure have been introduced [1, 12].

Catalys Laser has a liquid optics interface that does not need applanation of the cornea. The liquid patient interface has been associated with a lower IOP increase than the corneal applanation systems. Moreover, real-time IOP measurements assessed in porcine eyes underwent surgery with the Catalys platform were lower if recorded in the vitreous cavity instead of the aqueous humour [13].

Contact lens SoftFit™ interface of the LenSx platform has been recently introduced, as well. The soft contact lens, made up of a hydrogel material, closely matches the curvature of the cornea with minimal distortions, preserving the natural corneal curvature and fixing the eyeball. This type of interface gives higher control and stability reducing eye movements if compared to LOI, thus decreasing also the pressure to be employed on the eye by the surgeon [10].

At the beginning of the FLACS procedures, holding and manually placing the suction ring on the eye, sometimes without a lid speculum, can be very challenging in patients who have small palpebral fissures or difficult fixation. This manoeuvre is easier with LenSx due to the smaller diameter of the limbal suction ring [10].

In our work, the surgeon had a great experience with both lasers. A similar ease of the docking phase was found in both groups, likely due to high confidence of the experienced surgeon with different laser systems.

Similarly to our findings, the current SoftFit interface has been associated with an increase of IOP by approximately 16 mmHg, without short- or long-term complications directly related to the higher levels of IOP from the baseline [10].

Our results demonstrated a statistically significant transient increase in IOP from the baseline values in both patient interface systems, just after the docking phase and suction ring removal. Immediately after cataract surgery, IOP values decreased and came back to preoperative values at 1 day postoperatively.

Moreover, the rise in IOP during the suction phase was significantly higher in the LOI system versus the contact lens interface.

The highest levels of IOP induced by the vacuum using the soft lens interface was approximately 11 mmHg above the predocking IOP/the preoperative value, while in the LOI system, the vacuum-induced IOP rose by about 20 mmHg compared to baseline/compared to presurgery. Previous studies described very high IOP levels using flat and curved contact lenses for corneal flap creation with a femtosecond laser. Vetter et al. in their experimental study with enucleated porcine globes found much higher mean IOP levels than those reported in our work [7]. Talamo et al. [14] measured intraocular pressure ex vivo in porcine and human cadaver eyes and found a mean IOP rise of 32.4 mmHg ± 3.4 in the curved contact lens interface group and 17.7 mmHg ± 2.1 mmHg in the LOI group during the suction phase.

Schulz et al. [15], in a prospective clinical trial, found a mean IOP level of 27.7 mmHg ± 5.5, after removing the LOI interface, which is comparable to those obtained in our study in terms of amount of IOP increase in the liquid optics system.

On the contrary, we observed a lower rise in IOP with the SoftFit™ if compared with data reported in the literature regarding curved interfaces [7, 14]. It is also true that they considered rigid curved interfaces with a rigid contact lens, conversely to our work. The soft contact lens interface sets with more stability of the eye with a decreased risk for suction loss, differently from the rigid one that does not perfectly match the natural ocular shape, thus resulting in IOP rise [10, 14].

LOI has a mechanical contact only outside the limbus, on the overlying conjunctiva, where anatomic alterations are minimal, thus minimizing globe deformation and corneal irregularities. Anyway, LOI has been associated with pressure rises during the docking process because of the force downward of the suction ring by the additional weight of the disposable lens. In addition, micromovements of the eye could further increase the IOP during suction [13, 16].

We did not expect additional IOP increase from the BBS used because of the openings of the suction ring that can be a way of leak when the solution is displaced by the lens during docking.

Another interesting element to consider is the possibility of cavitation bubbles to cause an additional increase of IOP with their action of expansion of the lens volume and capsular bag [17].

Less energy to fragment a softer cataract could be correlated to a lower production of bubbles and a lower intraocular pressure. However, our sample included homogeneous grade of cataract, and we did not consider this aspect.

Our work has the unique angle of describing two completely different designs of interfaces that have shown to be associated with a minimal and transient increase in IOP, without any anatomical changes and any iatrogenic damage to the optic disc, confirming the safety of FLACS with excellent final anatomical and functional outcomes and no IOP-related postoperative complications.

Sudden increases of IOP, even though not very high, can be dangerous for the ocular structure [8]. Anyway, in our study, although a rise of IOP during suction was registered immediately after the suction procedure, IOP levels decreased immediately after the docking phase and suction ring removal and IOP values at 1 and 7 days after the surgical procedure were not statistically significant different than preoperative values.

In a nonrandomized prospective case series, the authors reported a higher transient increase of IOP after vacuum undocking in glaucomatous subjects than in those without the disease, but it seemed to be well tolerated in the short period in both groups [8].

## 4. Conclusions

To the best of our knowledge, no other study has compared the IOP variations in vivo during FLACS comparing these two types of interface systems: the fluid-filled interface versus the current soft contact lens system.

The main limitations of our study are its retrospective nature and the lack of IOP readings during the laser treatment to better understand its effect on IOP changes. Ibarz et al. [13] measured IOP in porcine eyes, every five seconds during the femtosecond procedure using a direct cannulation system to the anterior chamber. However, the real-time IOP in porcine eyes did not show any effect of the laser treatment on IOP modifications, and we believe that the transient increase in IOP during the docking phase is probably related to the greater pressure of the LOI suction ring and of suction generated through the vacuum, independently from the action of the femtosecond laser itself.

## Figures and Tables

**Table 1:** Demographic and clinical characteristics at the baseline.

Patient demographics and preoperative clinical status					
Parameter	Group 1		Group 2		*p* value (<0.01)
	Mean ± SD	Range	Mean ± SD	Range	
Age (y)	72.4 ± 7.6	47, 89	71.5 ± 13.9	23, 89	0.69
Male : female (n)	33 : 28	—	27 : 28	—	—
Left eye : right eye (n)	37 : 24	—	32 : 23	—	—
ACD (mm)	3.06 ± 0.24	2.61, 3.6	3.17 ± 0.38	2.58, 4.06	0.42
CCT (*µ*m)	560.9 ± 35.6	525, 630	555.2 ± 28.5	509, 634	0.38
AL (mm)	23.84 ± 1.49	21.56, 28.9	23.92 ± 1.36	21.93, 27.3	0.76
Preop IOP (mmHg)	14.1 ± 0.4	10, 22	13.8 ± 0.4	10, 20	0.15

ACD: anterior chamber depth; CCT: central cornea thickness; AL: axial length.

**Table 2:** Mean IOP data at the different study time points of the two groups.

Mean IOP data at the different study time points							
Study time points	Group 1			Group 2			
	Mean ± SD (mmHg)	*p* value^1^ (<0.01)	*p* value^2^(<0.01)	Mean ± SD (mmHg)	*p* value^1^ (<0.01)	*p* value^2^ (<0.01)	*p* value^3^ (<0.01)
Before suction	14.1 ± 0.4	1.0	—	13.8 ± 0.4	1.0	—	0.68
After vacuum	33.2 ± 1.1	<0.001	—	24.2 ± 1.4	<0.001	—	<0.001
After phaco	21.4 ± 0.9	<0.001	<0.001	20.2 ± 1.2	<0.001	0.67	0.395
1 day after	14.0 ± 0.4	1.0	<0.001	12.7 ± 0.3	1.0	<0.001	0.01
7 days after	13.9 ± 0.3	1.0	<0.001	13.2 ± 0.3	1.0	<0.001	0.09

Before suction: IOP measured before laser treatment; after vacuum: IOP measured just after vacuum turned off and suction ring removed on lying patients; after phaco: IOP measured after cataract surgery; 1 day after: IOP measured at 1 day after surgery; 7 days after: IOP measured at 7 days after surgery. *p* value^1^: statistical significance of IOP values compared to preoperative values; *p* value^2^: statistical significance of IOP values compared to values obtained after suction phase; *p* value^3^: statistical significance of the IOP difference between the two groups at each study time points. Group 1: liquid optics interface. Group 2: SoftFit™ Patient Interface.

## Data Availability

All data will be available if requested to the corresponding author.
